# Non-exposure LECS (CLEAN-NET) for gastric cancer: long-term outcomes of a function-preserving strategy with selective nodal assessment

**DOI:** 10.1007/s00464-026-13139-z

**Published:** 2026-07-14

**Authors:** Manabu Onimaru, Haruhiro Inoue, Kenta Nakahara, Koji Otsuka, Yoshio Deguchi, Noboru Yokoyama

**Affiliations:** 1 Digestive Diseases Center, Showa Medical University Koto Toyosu Hospital, 5-1-38 Toyosu, Koto-Ku, Tokyo, 135-8577 Japan; 2 Digestive Diseases Center, Showa Medical University Northern Yokohama Hospital, Yokohama, Japan

**Keywords:** Gastric cancer, LECS, CLEAN-NET, Long-term outcomes, Function-preserving surgery

## Abstract

**Background:**

Non-exposure laparoscopic and endoscopic cooperative surgery (LECS), known as CLEAN-NET (combination of laparoscopic and endoscopic approaches to neoplasia with non-exposure technique), was developed for gastric submucosal tumors; however, its oncologic validity for gastric cancer remains unclear. We evaluated long-term oncologic outcomes of CLEAN-NET in patients with clinically diagnosed cT1N0 gastric cancer during the early developmental phase of this technique.

**Methods:**

We retrospectively analyzed 19 consecutive patients who underwent CLEAN-NET between December 2008 and June 2010. Treatment selection was based on preoperative clinical staging available at that time. Limited regional lymph node assessment was performed in selected cases, and pathological findings were classified according to the eCura system. Recurrence of the gastric cancer treated by CLEAN-NET, metachronous gastric cancer, overall survival, and causes of death were evaluated.

**Results:**

The median follow-up period was 6.2 years, with follow-up extending up to 16.8 years. Although treatment selection was based on preoperative cT1N0 assessment, 14 patients (73.7%) were subsequently classified as eCuraC-2 because of postoperative pathological risk factors. Limited regional lymph node dissection was performed in 15 patients, and pathological lymph node metastasis was identified in 1 patient. Additional surgery was performed in 3 patients. No recurrence of the gastric cancer treated by CLEAN-NET was observed. Metachronous gastric cancer developed in the preserved stomach in 2 patients, including 1 death from metachronous gastric cancer. During follow-up, 3 patients died: 1 from lung cancer, 1 from metachronous gastric cancer, and 1 from pneumonia.

**Conclusion:**

This historical cohort provides rare ultra-long-term observational data after CLEAN-NET for gastric cancer. Although no recurrence of the gastric cancer treated by CLEAN-NET was observed, the findings do not establish CLEAN-NET as an alternative to guideline-recommended gastrectomy. Rather, they may inform future studies of function-preserving, non-exposure local resection combined with validated nodal navigation strategies.

**Graphical Abstract:**

**Figure:**
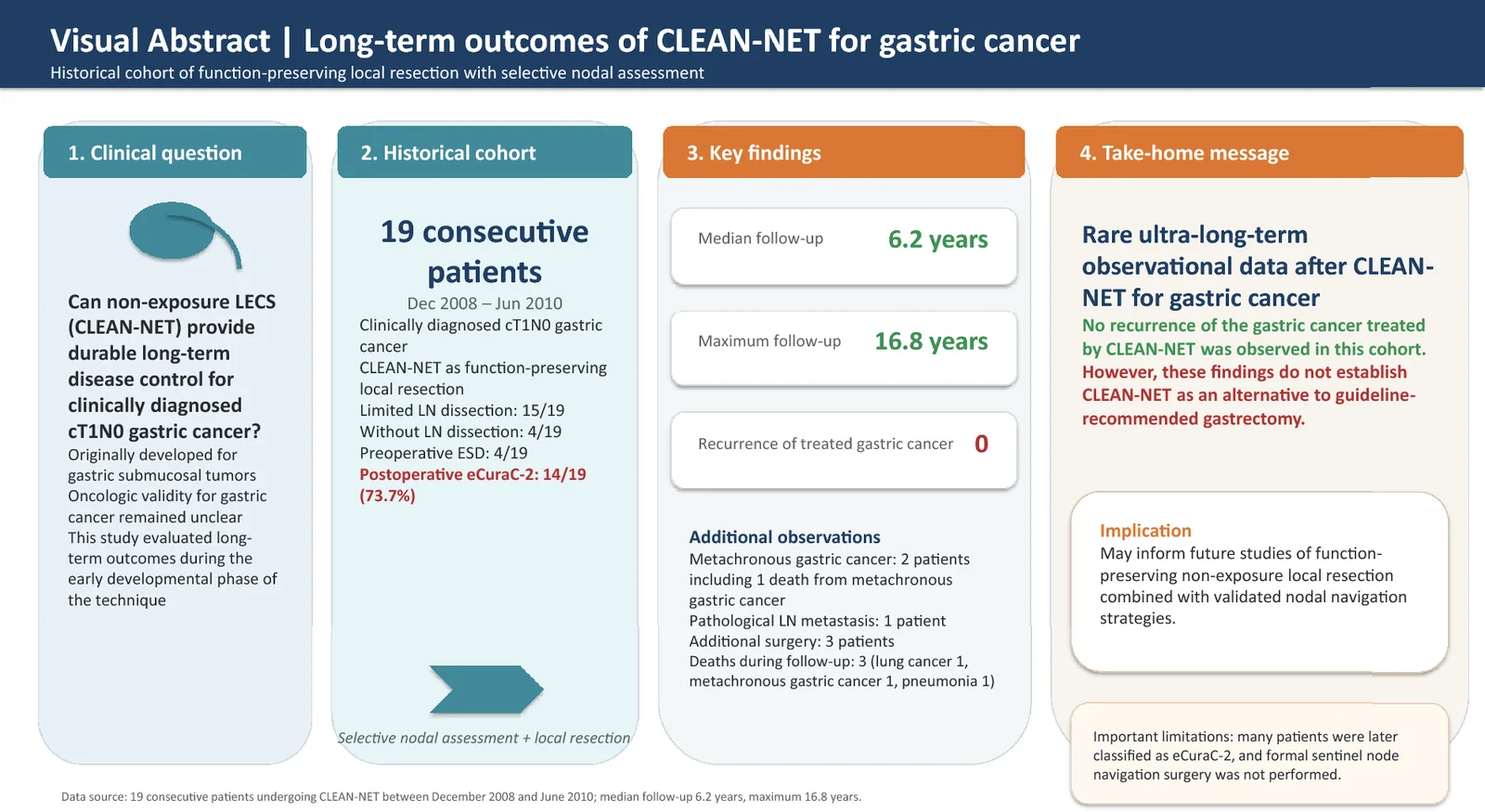

Gastric cancer remains one of the leading causes of cancer-related mortality worldwide [1]. For early-stage disease, endoscopic submucosal dissection (ESD) and standard gastrectomy with systematic D2 lymphadenectomy represent established treatment options, depending on the risk of lymph node metastasis [2]. While ESD enables organ preservation in carefully selected patients, its indications are restricted by strict oncologic criteria, particularly due to the risk of lymph node metastasis [3–5]. Conversely, standard gastrectomy with D2 lymphadenectomy provides reliable oncologic clearance but is associated with functional impairment and surgical morbidity [6, 7].

Laparoscopic and endoscopic cooperative surgery (LECS) was developed as a hybrid approach to overcome the limitations of both endoscopic and surgical treatments. By combining endoscopic visualization with laparoscopic resection, LECS enables precise local resection while preserving gastric anatomy [8]. However, the original LECS technique involves intentional opening of the gastric wall, raising significant oncologic concerns regarding tumor cell dissemination and intraperitoneal contamination when applied to gastric cancer [9].

To address this limitation, non-exposure techniques such as Combination of Laparoscopic and Endoscopic Approaches to Neoplasia with Non-Exposure Technique (CLEAN-NET) were developed [10, 11]. This approach allows full-thickness resection without opening the gastric lumen, thereby potentially reducing the risk of tumor spillage while preserving gastric function [9]. Although LECS and its modifications have been reported for gastric tumors, including early gastric cancer, available studies have primarily focused on technical feasibility and short- to mid-term outcomes [12–14].

However, robust long-term oncologic validation of LECS-type local resection for gastric cancer remains lacking. In particular, long-term outcomes beyond 10 years have rarely been systematically reported. Clarification of the long-term clinical course after non-exposure LECS is therefore important, especially as function-preserving strategies and sentinel node navigation concepts continue to evolve.

The present study evaluates the long-term outcomes of patients who underwent non-exposure LECS (CLEAN-NET) for gastric cancer during its early developmental phase. With follow-up extending up to 16.8 years, this study provides rare ultra-long-term observational data on the clinical course after this function-preserving approach.

## Materials and methods

### Study design and patients

This retrospective study included consecutive patients who underwent non-exposure LECS (CLEAN-NET) for gastric cancer between December 2008 and June 2010 at Showa Medical University Northern Yokohama Hospital, Yokohama, Japan. All procedures were performed by the same surgical team responsible for the present analysis. This period corresponded to the initial clinical application of CLEAN-NET for malignant gastric lesions.

During this early phase, CLEAN-NET was applied to patients with clinically early-stage gastric cancer, primarily cT1N0, based on the preoperative assessment available at that time. Preoperative evaluation included conventional endoscopy, biopsy histology, assessment of macroscopic morphology and ulcerative findings, computed tomography, and endoscopic ultrasonography when available. Patients with radiologic evidence of lymph node or distant metastasis were not considered candidates for local resection.

Treatment selection was based on tumor-related factors, including clinical depth, tumor size and location, macroscopic morphology, biopsy histology, and technical feasibility of ESD, as well as patient-related factors such as age, comorbidity, and patient preference. CLEAN-NET was selected for lesions considered technically difficult for ESD according to the standards at that time, for selected patients requiring additional surgical management after non-curative ESD, or for patients in whom an organ-preserving approach was considered clinically desirable.

## Surgical procedure

**Fig. 1:**
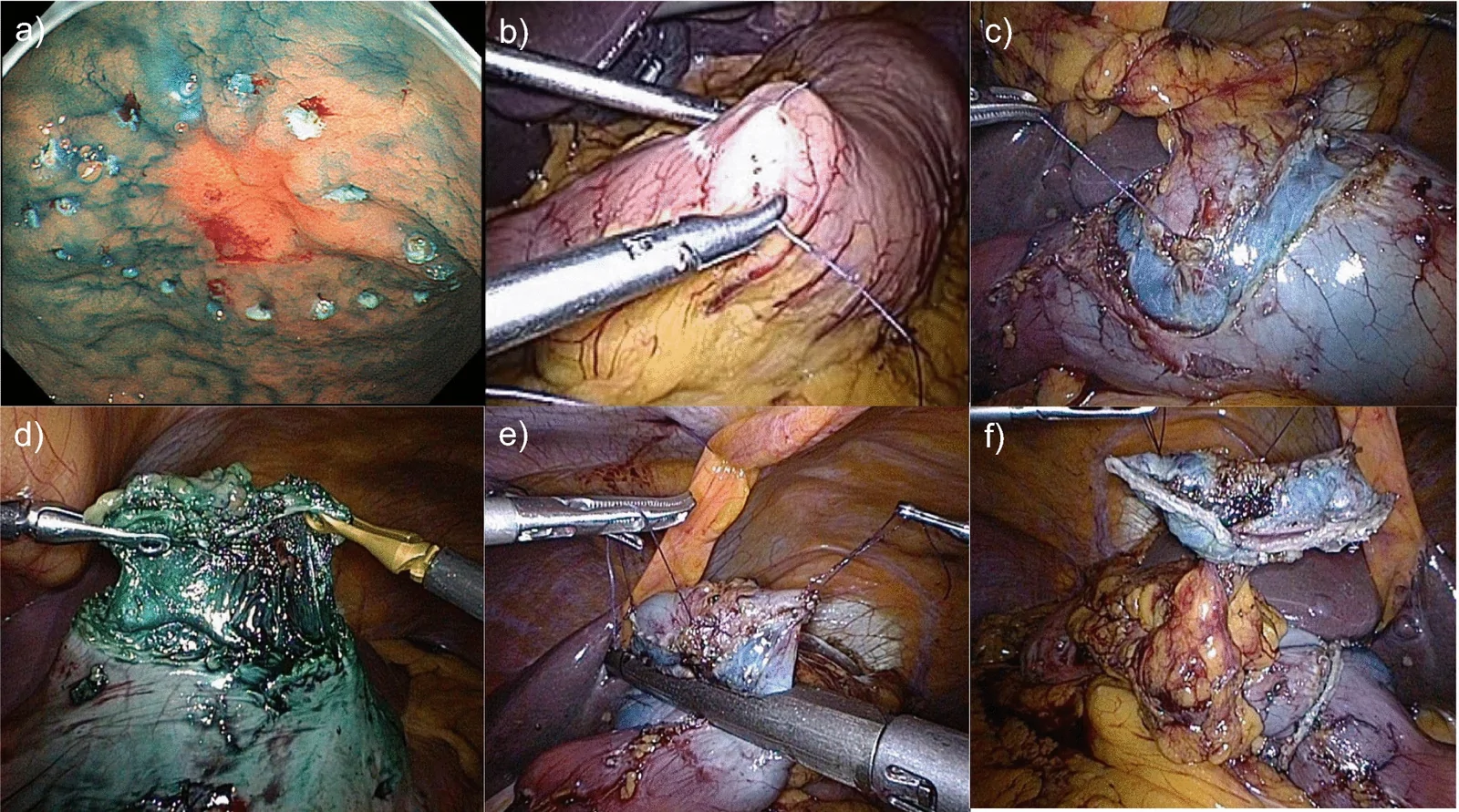
Surgical procedure of non-exposure LECS (CLEAN-NET). **a** Endoscopic marking around the lesion. **b** Placement of traction sutures to elevate the tumor. **c** Seromuscular incision along the marked line. **d** Elevation of the tumor toward the abdominal cavity. **e** Full-thickness resection of the gastric wall using a linear stapler. **f** En bloc retrieval of the specimen with limited regional lymph node dissection

CLEAN-NET was performed as previously described [10, 11]. The key procedural steps are illustrated in Fig. 1. Briefly, tumor location was identified endoscopically, and laparoscopic full-thickness resection was carried out without intentional opening of the gastric lumen. The lesion was resected with adequate margins under combined laparoscopic and endoscopic guidance, and the specimen was retrieved without intraperitoneal exposure of the tumor.

In selected cases, indocyanine green (ICG) was injected locally around the tumor to visualize lymphatic flow. Limited regional lymph node dissection was performed according to tumor location and intraoperatively observed lymphatic drainage, mainly within the relevant perigastric nodal basin. This procedure was not based on a standardized D2 template and did not constitute formal sentinel node navigation surgery.

Systematic D2 lymphadenectomy was not routinely performed at the initial procedure. In selected patients, additional surgery was considered following pathological evaluation of the resected specimen.

## Pathological evaluation

Resected specimens were evaluated according to the Japanese Classification of Gastric Carcinoma [15]. Tumor size, histological type, depth of invasion, ulcerative findings, lymphatic invasion, venous invasion, and horizontal and vertical resection margins were recorded. The eCura classification was assigned based on these pathological findings according to the established criteria for curability after endoscopic resection for early gastric cancer [16, 17]. Lymphatic and venous invasion were recorded as ly0/ly1 and v0/v1, respectively. In patients who underwent lymph node dissection, the number of retrieved lymph nodes and pathological nodal status were recorded.

## Follow-up and oncologic outcomes

Patients were followed postoperatively with periodic clinical examination, endoscopic surveillance, and imaging studies according to institutional practice. Postoperative outcomes were evaluated by distinguishing recurrence of the gastric cancer treated by CLEAN-NET from metachronous gastric cancer arising in the preserved stomach. Recurrence was defined as radiologic or histologic evidence of relapse of the gastric cancer treated by CLEAN-NET. Metachronous gastric cancer was recorded separately and was not classified as recurrence. Overall survival (OS) was calculated from the date of CLEAN-NET to the date of death or last follow-up.

## Ethics statement

This retrospective study was approved by the Institutional Review Board of Showa Medical University Koto Toyosu Hospital, Tokyo, Japan (Approval No. C-T2026-0341). The requirement for informed consent was waived due to the retrospective nature of the study**.**

## Statistical analysis

Continuous variables are presented as median (range), and categorical variables as number (percentage). OS was estimated using the Kaplan–Meier method. Recurrence of the gastric cancer treated by CLEAN-NET and metachronous gastric cancer were summarized as clinical events. Given the small sample size, no comparative statistical testing was performed. Analyses were performed using JMP version 19 (SAS Institute Inc., Cary, NC, USA).

## Results

### Patient cohort and perioperative outcomes

**Fig. 2:**
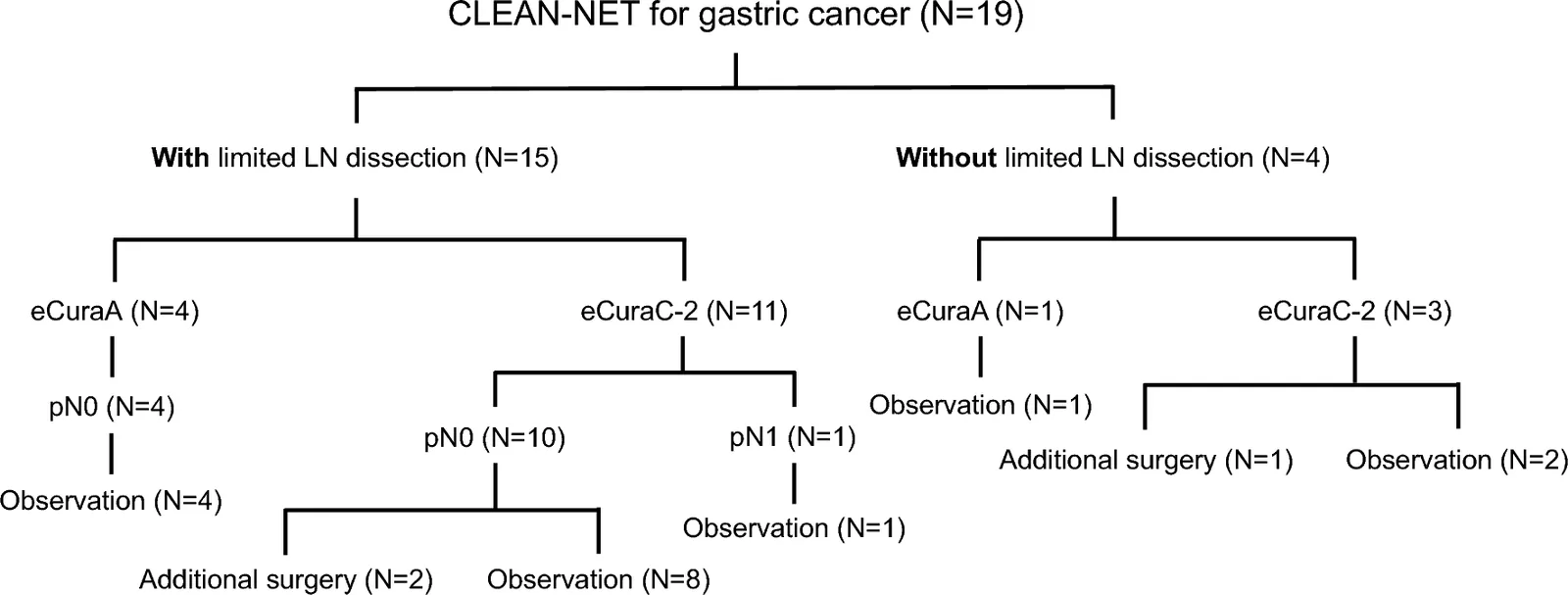
Patient flow after CLEAN-NET for gastric cancer. Nineteen patients underwent CLEAN-NET, of whom 15 received limited lymph node (LN) dissection and 4 did not. Limited LN dissection was performed according to tumor location and intraoperative lymphatic drainage, mainly within the relevant perigastric nodal basin. Pathological classification according to the eCura system, nodal status, and subsequent management, including additional surgery or observation, are shown

A total of 19 patients underwent CLEAN-NET for gastric cancer during the study period (Fig. 2). Baseline patient characteristics and perioperative outcomes are summarized in Table 1. The median age was 68 years (range, 45–82), and 16 patients (84.2%) were male. Fifteen patients (78.9%) underwent CLEAN-NET with limited lymph node dissection, whereas 4 patients (21.1%) underwent CLEAN-NET without lymph node dissection. Preoperative endoscopic submucosal dissection had been performed in 4 patients (21.1%).

**Table 1:**
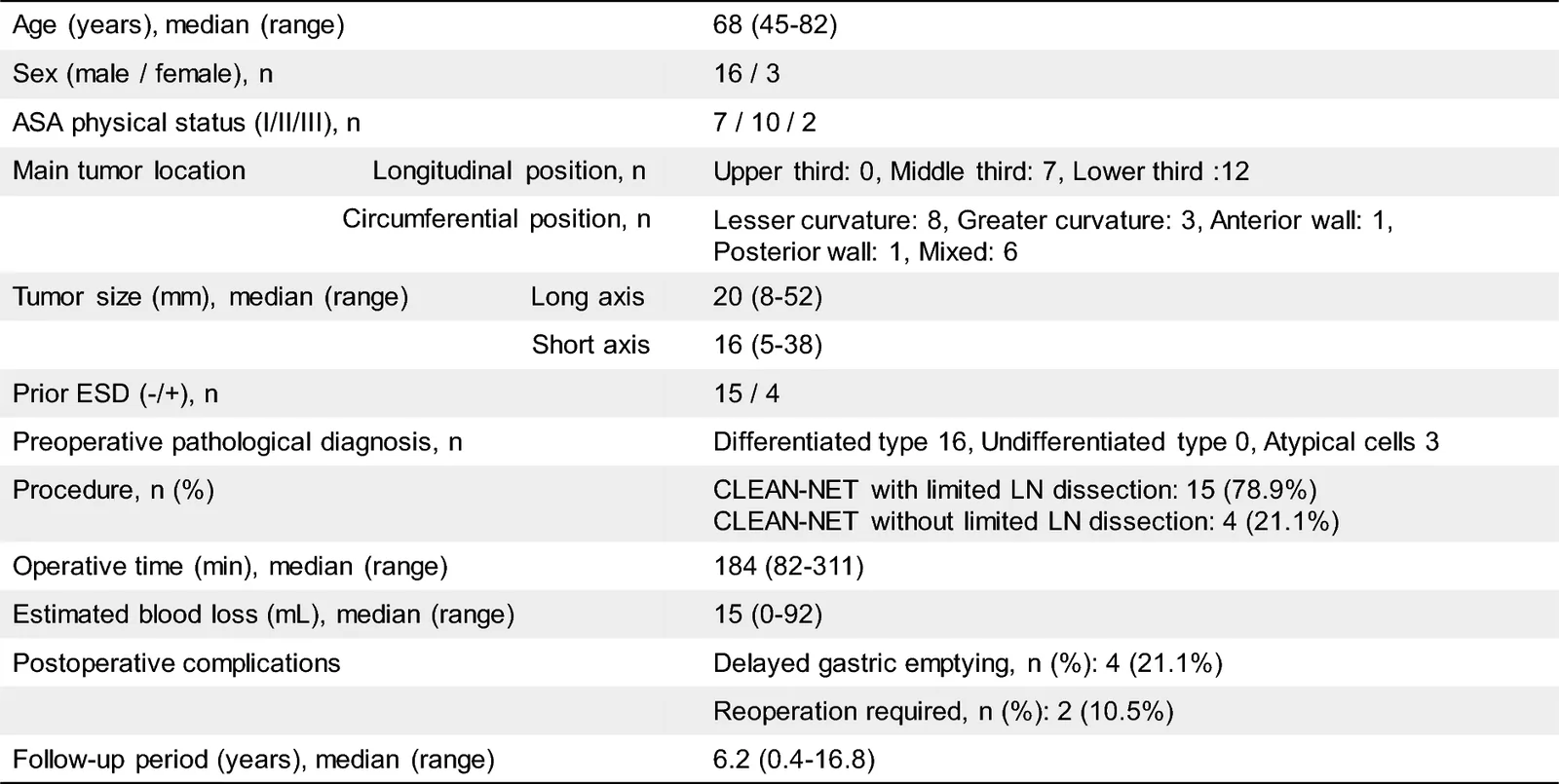
Baseline patient characteristics and perioperative outcomes (*N* = 19)

Values are presented as median (range) or number (%), unless otherwise indicated

*ASA* American Society of Anesthesiologists, *ESD* Endoscopic submucosal dissection, *LN* Lymph node. “Mixed” indicates a lesion involving more than one circumferential aspect of the stomach

The median operative time was 184 min (range, 82–311), and the median estimated blood loss was 15 mL (range, 0–92). Postoperative delayed gastric emptying occurred in 4 patients (21.1%), and reoperation was required in 2 patients (10.5%). The median follow-up period was 6.2 years (range, 0.4–16.8 years), with follow-up extending up to 16.8 years.

## Pathological findings and eCura classification

Although all patients were clinically assessed as cT1N0 before surgery, pathological evaluation demonstrated a higher-risk profile in many cases. Pathological examination revealed eCuraA in 5 patients and eCuraC-2 in 14 patients (Table 2). The eCuraC-2 classification was assigned because of postoperative pathological risk factors, including deeper invasion and/or lymphovascular invasion. In selected cases, pathological evaluation demonstrated invasion deeper than expected preoperatively. Lymphatic or vascular invasion was observed in several eCuraC-2 patients. Negative horizontal and vertical margins (HM0/VM0) were achieved in all cases. Limited lymph node dissection was performed at the time of initial CLEAN-NET in 15 patients (78.9%).

**Table 2:**
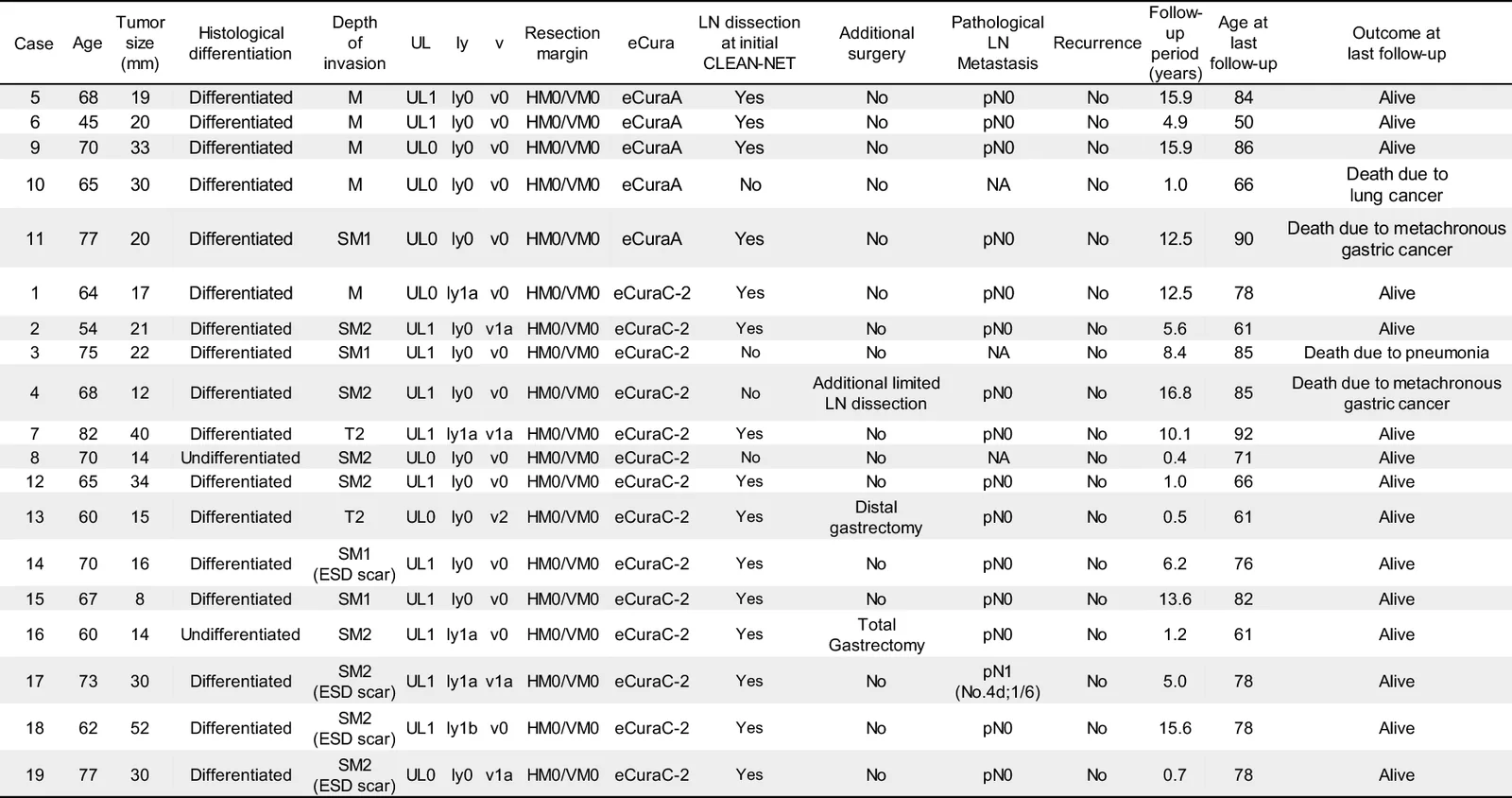
Clinicopathological characteristics and long-term outcomes according to eCura classification

*UL* Ulcerative findings, *ly* lymphatic invasion, *v* venous invasion, *HM* Horizontal margin, *VM* Vertical margin, *LN* Lymph node, *NA* Not applicable

## Lymph node status and additional surgery

Among the 15 patients who underwent lymph node dissection at the initial CLEAN-NET, pathological lymph node metastasis was detected in only 1 patient, involving a single No. 4d lymph node (1/6 nodes examined) (6.7%). This lymph node metastasis was identified at the time of initial CLEAN-NET, and the patient did not undergo additional surgery. The patient has remained free from recurrence of the gastric cancer treated by CLEAN-NET during follow-up.

Additional surgery was performed in 3 patients based on the pathological findings after CLEAN-NET: one patient underwent additional limited lymph node dissection alone, one underwent distal gastrectomy, and one underwent total gastrectomy. All additional procedures were carried out approximately two months after confirmation of the CLEAN-NET pathological diagnosis. No residual nodal disease was identified in the resected specimens.

## Visualization of long-term clinical course

**Fig. 3:**
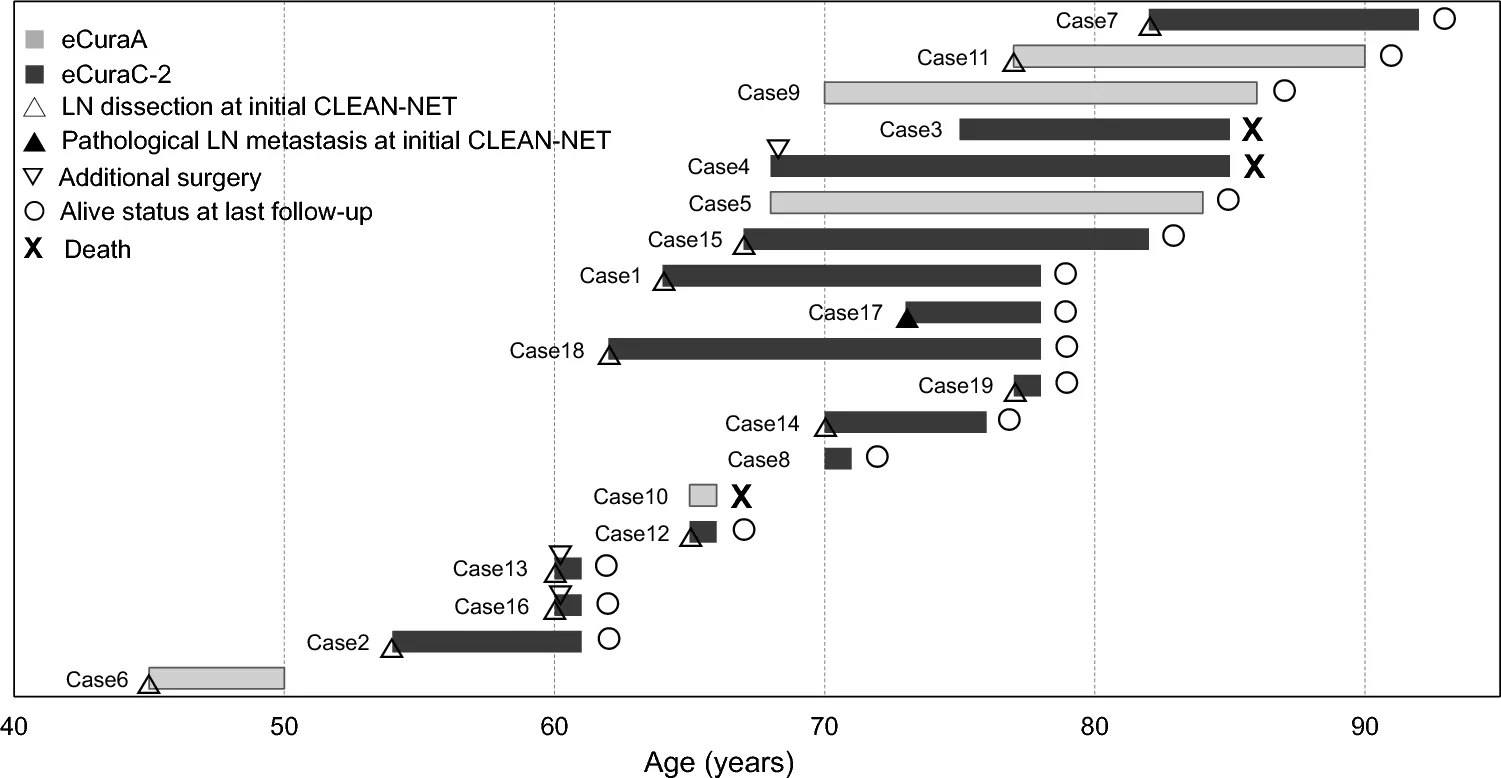
Swimmer plot illustrating long-term age-based clinical course according to eCura classification. Each horizontal bar represents an individual patient from age at CLEAN-NET to age at last follow-up or death. Bar color indicates eCura classification. Symbols indicate lymph node dissection at initial CLEAN-NET (△); pathological lymph node metastasis (▲); additional surgery (▽); alive status at last follow-up (○); and death (X). The plot demonstrates the long-term follow-up period and event timing for each patient

The swimmer plot illustrates the long-term clinical course of individual patients according to eCura classification (Fig. 3). Each horizontal bar represents an individual patient from the age at CLEAN-NET to the age at last follow-up or death. Bar color indicates eCura classification. Symbols indicate lymph node dissection at initial CLEAN-NET, pathological lymph node metastasis, additional surgery, survival at last follow-up, and death. This visualization was used to illustrate both the duration of follow-up and the timing of relevant clinical events in each patient.

## Long-term oncologic outcomes

No recurrence of the gastric cancer treated by CLEAN-NET was observed during the entire follow-up period, despite the high proportion of eCuraC-2 lesions. Two patients developed metachronous gastric cancer in the preserved stomach. One of these patients died of metachronous gastric cancer, whereas the other remained alive after treatment. Because metachronous gastric cancer was considered a new lesion arising in the preserved stomach, it was recorded separately from recurrence of the gastric cancer treated by CLEAN-NET.

**Fig. 4:**
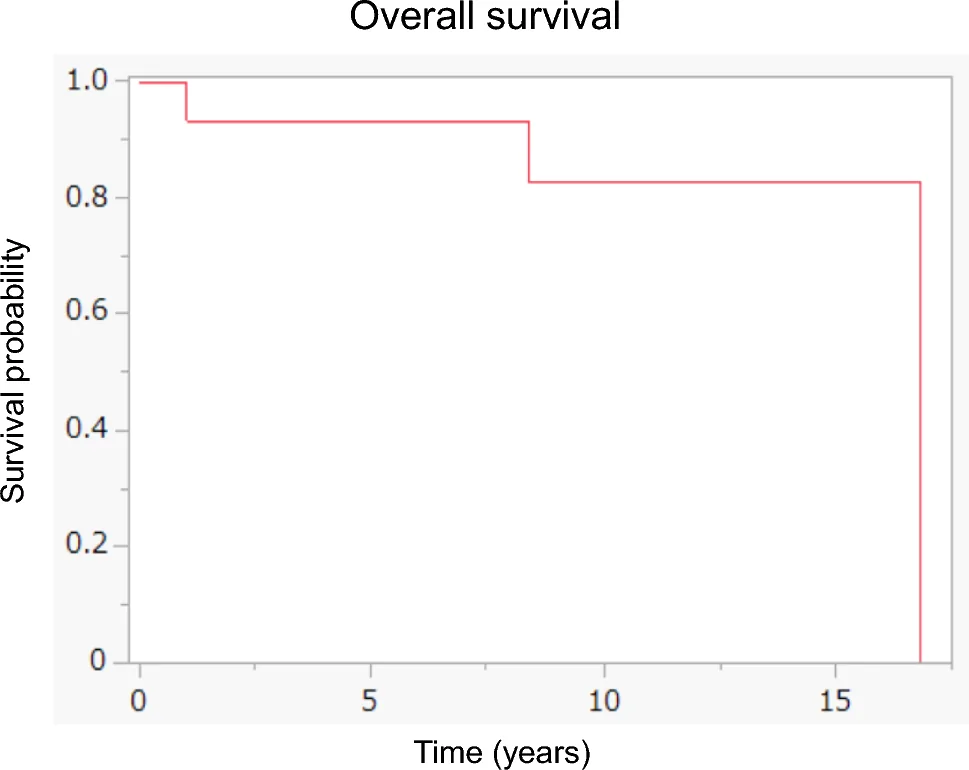
Kaplan–Meier curve of overall survival after CLEAN-NET. Overall survival was calculated from the date of CLEAN-NET to the date of death or last follow-up. Three deaths occurred during follow-up, including one from lung cancer, one from metachronous gastric cancer, and one from pneumonia

During follow-up, three patients died: one from lung cancer, one from metachronous gastric cancer, and one from pneumonia. Overall survival is shown in Fig. 4.

## Discussion

This study provides rare long-term observational data after non-exposure LECS (CLEAN-NET) for gastric cancer, with follow-up extending up to 16.8 years. No recurrence of the gastric cancer treated by CLEAN-NET was observed. However, metachronous gastric cancer occurred in the preserved stomach, including one fatal case, and the absence of recurrence should therefore be interpreted specifically in relation to the lesion treated by CLEAN-NET.

The discrepancy between preoperative cT1N0 assessment and postoperative eCuraC-2 classification is central to the interpretation of this study. Treatment selection was based on the best available preoperative clinical assessment during the early developmental phase of CLEAN-NET, whereas the eCura classification was assigned postoperatively using definitive histopathological findings. Thus, the high proportion of eCuraC-2 lesions should not be interpreted as intentional selection of pathologically high-risk lesions for local resection, but rather as a consequence of postoperative pathological upstaging and risk reclassification, reflecting the limitations of preoperative risk prediction at that time.

The present cohort reflects an early developmental phase of minimally invasive gastric cancer surgery. At that time, ESD had not yet reached its current level of technical refinement, and laparoscopic D2 gastrectomy was still evolving. Under these circumstances, LECS-based laparoscopic local resection was selected for early gastric cancers that were technically difficult to manage endoscopically according to prevailing standards, as well as for selected post-ESD cases in which an organ-preserving surgical approach was considered desirable.

CLEAN-NET was developed as a modification of conventional LECS to avoid gastric wall opening. By enabling full-thickness resection without exposure of the gastric lumen to the peritoneal cavity, this technique represents a non-exposure full-thickness resection strategy that may potentially reduce the risk of intraperitoneal tumor cell dissemination while maintaining gastric preservation. However, CLEAN-NET alone does not address the risk of lymph node metastasis, which remains the central oncologic issue when applying local resection to gastric cancer.

It should also be acknowledged that perioperative outcomes during this early developmental phase differed from contemporary standards. Postoperative delayed gastric emptying occurred in four patients (21.1%) and two required reoperation. These outcomes should be interpreted within the context of the early developmental period of the technique rather than current practice.

Importantly, the absence of recurrence should not be overinterpreted. Only a limited number of patients had a meaningful residual risk of recurrence and were observed without additional gastrectomy. Given the limited sample size, the absence of recurrence in such a small subgroup cannot establish oncologic safety or equivalence to standard gastrectomy, but should be regarded as descriptive long-term observational data from a historical cohort.

In contemporary practice, patients with pathological nodal metastasis or pT2 disease would generally be considered for additional gastrectomy with systematic lymphadenectomy. The fact that additional surgery was not performed in all such patients in the present series reflects the historical and individualized nature of this early cohort and should not be interpreted as support for omitting standard additional surgery today.

The clinical value of the non-exposure concept differed between primary CLEAN-NET cases and post-ESD cases. In primary CLEAN-NET cases, non-exposure full-thickness resection was intended to avoid intraperitoneal tumor exposure. In post-ESD cases, the primary lesion had already been removed endoscopically; therefore, the value of the non-exposure concept was more limited. In these cases, CLEAN-NET was used as a LECS-based organ-preserving surgical approach for additional local or regional treatment after non-curative ESD.

Limited regional lymph node dissection was performed in 15 patients, and pathological metastasis was identified in only 1 case (6.7%), involving a single No. 4d node. This patient did not undergo subsequent gastrectomy and has remained free from recurrence of the gastric cancer treated by CLEAN-NET. Although definitive conclusions cannot be drawn from a single observation, this finding raises the possibility that limited nodal clearance may have contributed to disease control.

A major limitation of this study is that formal sentinel node navigation surgery was not performed. Although ICG-assisted selective regional nodal assessment was used in selected patients, the procedure was not standardized and did not include validated sentinel basin mapping or intraoperative confirmation of sentinel node negativity. Therefore, the present findings should not be extrapolated to contemporary sentinel node navigation surgery or used to justify local resection without validated nodal assessment. Future studies should incorporate validated nodal navigation strategies when evaluating non-exposure local resection for gastric cancer.

In the current treatment landscape, ESD remains the preferred organ-preserving treatment for lesions that meet established curability criteria, whereas minimally invasive gastrectomy with systematic lymphadenectomy remains the standard approach when nodal risk is clinically relevant. Compared with ESD, CLEAN-NET enables full-thickness local resection but does not by itself address the risk of lymph node metastasis. Compared with gastrectomy, CLEAN-NET may better preserve gastric anatomy, but provides less comprehensive nodal clearance. Therefore, the contemporary role of CLEAN-NET in gastric cancer is likely limited to highly selected clinical scenarios or future protocols combined with validated nodal navigation.

No recurrence of the gastric cancer treated by CLEAN-NET was observed in this cohort. However, two patients developed metachronous gastric cancer in the preserved stomach, including one death from metachronous gastric cancer. This distinction is important because the absence of recurrence refers only to the lesion treated by CLEAN-NET and should not be interpreted as absence of subsequent gastric cancer risk. Because CLEAN-NET preserves the stomach, patients remain at risk for metachronous gastric cancer arising from the background mucosa. Potential risk factors for metachronous gastric cancer include Helicobacter pylori infection, atrophic gastritis, intestinal metaplasia, and synchronous or multiple gastric neoplasia [18, 19]. Although these factors were not systematically evaluated in this historical cohort, annual endoscopic surveillance may be a reasonable minimum strategy after organ-preserving local resection, with shorter intervals considered for high-risk patients.

Another clinically relevant observation concerns patient age. Several individuals were followed into advanced age, including patients beyond 90 years old, without death attributable to recurrence of the gastric cancer treated by CLEAN-NET. In contrast, three patients died of other causes or metachronous disease during long-term follow-up. These data highlight the reality that, particularly in elderly populations, competing mortality risks may outweigh the risk of recurrence from the treated lesion. In such settings, balancing oncologic benefit against surgical invasiveness becomes especially important.

Clinical practice has evolved considerably since the early period represented in this study. ESD is now technically mature, and standard gastrectomy with systematic D2 lymphadenectomy is routinely performed—often via minimally invasive laparoscopic approaches—with acceptable morbidity [20, 21]. Accordingly, when standard D2 gastrectomy is indicated to achieve guideline-recommended oncologic clearance, the role of CLEAN-NET in gastric cancer is likely limited. Importantly, these findings should not be interpreted as a substitute for guideline-recommended D2 gastrectomy when oncologically indicated.

The small sample size and retrospective design limit definitive conclusions regarding long-term oncologic safety. Additional limitations include the historical nature of the cohort, heterogeneous indications, non-standardized nodal assessment, lack of formal sentinel node navigation surgery, and the small number of patients with residual recurrence risk who were observed without additional gastrectomy. These findings should therefore be interpreted as hypothesis-generating rather than practice changing. Although limited by its small sample size and retrospective design, the exceptionally long follow-up provides unique insight into the long-term behavior of this approach.

This early historical series provides rare ultra-long-term observational data on CLEAN-NET for gastric cancer. No recurrence of the gastric cancer treated by CLEAN-NET was observed during follow-up of up to 16.8 years; however, metachronous gastric cancer occurred in the preserved stomach, including one death. Therefore, these findings should not be interpreted as establishing CLEAN-NET as an alternative to guideline-recommended gastrectomy. Rather, they provide hypothesis-generating data that may inform future studies of non-exposure local resection combined with validated nodal navigation strategies in highly selected patients.
